# The progress and challenges of circRNA for diabetic foot ulcers: A mini-review

**DOI:** 10.3389/fendo.2022.1019935

**Published:** 2022-11-30

**Authors:** Deer Li, Jiaxing Guo, Xiyu Ni, Guanwen Sun, Huhe Bao

**Affiliations:** ^1^ Graduate School, Inner Mongolia Medical University, Hohhot, China; ^2^ Department of Traumatology and Orthopedics, Inner Mongolia People’s Hospital, Hohhot, China; ^3^ Department of Joint Surgery, The Second Affiliated Hospital, Inner Mongolia Medical University, Hohhot, China

**Keywords:** diabetic foot ulcers, circRNAs, differential expression, diagnostic markers, therapeutic targets

## Abstract

Since the Human Genome Project was successfully completed, humanity has entered a post-genome era, and the second-generation sequencing technology has gradually progressed and become more accurate. Meanwhile, circRNAs plays a crucial role in the regulation of diseases and potential clinical applications has gradually attracted the attention of physicians. However, the mechanisms of circRNAs regulation at the cellular and molecular level of diabetic foot ulcer (DFU) is still not well-understood. With the deepening of research, there have been many recent studies conducted to explore the effect of circRNAs on DFU. In this mini-review, we discuss the potential role of circRNAs as therapeutic targets and diagnostic markers for DFU in order to gain a better understanding of the molecular mechanisms that underlie the development of DFU and to establish a theoretical basis for accurate treatment and effective prevention.

## Introduction

Diabetic foot ulcer (DFU) is one of the most common lower limb complications of diabetes mellitus (DM) (1). According to relevant studies, the 5-year and 10-year mortality of DFU patients is 22% and 71% respectively, and the amputation rate is 29.3% (2). The etiology of DFU is attributed to a variety of causes, including chronic inflammation, diabetic peripheral neuropathy, and vascular endothelial damage of the distal arterial vasculature. However, the lack of typical signs and symptoms in the early stages of the disease makes misdiagnosis and under diagnosis a common occurrence in clinical work. Although there are many medical and surgical treatments in clinic, the risk of chronic ulcer or even amputation is easy to occur due to the lack of effective diagnostic markers and treatment targets. Therefore, it is important to explore the etiological mechanism to provide theoretical basis for accurate treatment and diagnosis.

CircRNAs are important regulators in the cellular life cycle. It is a class of non-coding RNA formed by covalent cyclization, which is abundantly expressed in eukaryotic organisms and has high stability. Studies have shown that circRNAs play an important role in cell proliferation, apoptosis, metabolism, inflammation and other biological processes (3). In addition, circRNAs are closely related to the development of many diseases, such as DM and cancer (4). Therefore, this mini-review provides a comprehensive review of the biological role of circRNAs in DFU and explores the possibility of circRNAs as a therapeutic target and diagnostic marker for DFU by reviewing the literature and related materials.

## Pathophysiology of DFU

Normally, wound healing undergoes several phases, including hemostasis, inflammation, proliferation, migration, re-epithelization and remodeling (5). However, there are several factors that contribute to the non-healing of DFU. Hyperglycemia, chronic inflammation, dysfunction of microcirculation and macrocirculation, hypoxia, sensory neuropathy and neuropeptide signal damage are the main factors that lead to the difficulty of wound healing (6). However, hyperglycemia may be the most critical point of non-healing of wounds. It has been reported that hyperglycemia can promote vascular endothelial cell (ECs) dysfunction and induce apoptosis (7). ECs dysfunction leads to a decrease in various angiogenic and vasoactive factors secreted by it, as well as a decrease in new blood vessel formation (8–10). Studies have shown that endothelial dysfunction is the intrinsic cause of impaired wound healing (11–13). In addition, hyperglycemia directly affects the activity of keratinocytes (HEKs) and fibroblasts (FBs), leading to changes in protein synthesis, proliferation, and migration (14). It will seriously affect the re-epithelization and remodeling of the wounds, which in turn leads to non-healing of wounds. Recently, there is increasing evidence that hyperglycemia leads to impaired cell response to hypoxia (15). Hypoxia can prolong the damage by increasing the level of free oxygen radicals. Meanwhile, inflammation caused by chronic hyperglycemia will further increase oxidative stress and pro-inflammatory chemokines to reduce cell proliferation and migration then ultimately delay wound healing (16, 17). Studies have shown that diabetic peripheral neuropathy is one of the main causes of foot ulcers (18). It is believed that glial cell apoptosis and autophagy in the peripheral nervous system caused by hyperglycemia stimulation are the main causes for the occurrence and development of peripheral neuropathy (19–21). In brief, the main characteristics of diabetic wound non-healing are decreased angiogenesis, decreased recruitment of bone marrow-derived endothelial progenitor cells (EPC), decreased proliferation and migration of FBs and HEKs, and apoptosis of nerve cells (22). Interestingly, recent studies have shown that circRNAs play an important role in the pathophysiology of DFU wound healing. This may provide a novel idea for finding diagnostic marks and therapeutic targets of DFU.

## CircRNA

### Overview of circRNA molecules

**Figure 1:**
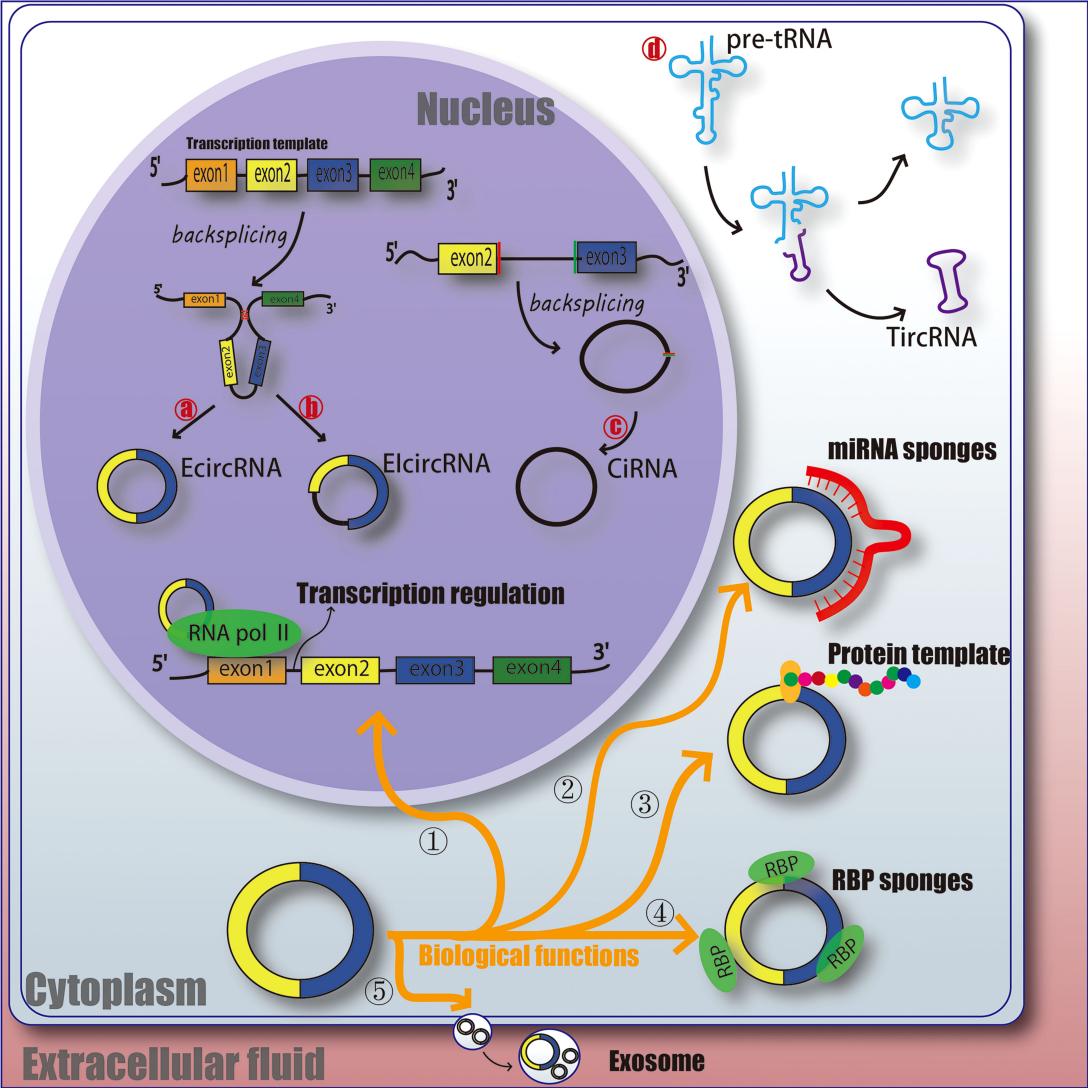
The function and classification of circRNA. EcircRNA, exonic circRNA. EIcircRNA, exon - intron circRNA. CiRNA, intronic circRNA. TircRNA, tRNA intronic circRNA. RNA pol II, RNA polymerase II. RBP, RNA-binding protein. miRNA, microRNA.

CircRNAs is a class of non-linear RNA molecule, which is not easily degraded by nucleic acid exonucleases and is formed by covalent cyclization. circRNAs are mainly produced by covalent connection of upstream and downstream sites during back-splicing. Most of the circRNAs were earlier undetected in RNA sequence due to the lack of 3 ‘ poly tails, and as the technology has evolved, over 183,000 have been identified now (23). Broadly, circRNAs can be classified into 4 types, EcircRNA composed of exons and mainly located in the cytoplasm; EIcircRNA with the combination of introns and exons and mainly located in the nucleus; CiRNA composed of introns, mainly located in the nucleus (24–27); circRNA produced by cyclization of viral RNA gene, tRNA, rRNA or snRNA (**Figure 1**).

### The main biological functions of circRNA

Understanding the ways which circRNAs participates in regulating biological processes further broadens our horizons. circRNAs has various biological functions, such as affecting the splicing of linear RNA and regulating transcription. ① Acting as a transcriptional regulator; CircRNAs can interact with U1 snRNA to form circRNA-U1 snRNP complex, and then further interact with RNA Pol II transcription complex at the promoter of parent gene to alter gene transcription and expression (28). For example, circ_ANKRD52, generated from gene ANKRD52, is capable of accumulating to its transcription sites and regulates elongation Pol II machinery acting as a positive regulator for transcription (29). ② Acting as miRNA sponges; MiRNA can bind directly to target mRNA in a base-pair fashion and trigger cleavage of mRNA or inhibit translation of mRNA (30). CircRNAs located in cytoplasm also contain complementary miRNA binding sites, and thus serve as competitive inhibitors for miRNA. In human cells, circ_ASAP1 can act as a sponge of miR_326 and miR_532_5p to promote hepatocellular carcinoma under hypoxic conditions (31). In addition, new circRNA sponges are continually being discovered in various disciplines. Circ_ITCH sponges miR-7, miR-17 and miR-214 in esophageal squamous cell carcinoma (ESCs) and inhibits tumor proliferation (32). CiRS-7 acts as a miR-7 sponge in many pathophysiological processes, including myocardial infarction, hepatocellular carcinoma (HCC) and gastric cancer (GC) (32–34). These results suggest that circRNAs as miRNA sponges might be a common function of circRNAs. ③ Protein templates; At first, people thought that circRNAs was a non-coding RNA (24, 35). However, recent studies have found that extensive N6-methyladenosine (m6A) modification is enough to drive circRNAs to translate in a cap-independent manner, as well as m6A reader YTHDF3 and translation initiation factors eIF4G2 and eIF3A (36). And further analysis indicates that translatable circRNAs may be common in human transcriptome (37). However, the translation ability and efficiency of circRNA are still remains controversial. ④ Binding with RBP (RNA-binding protein); CircRNAs have also been reported to act as sponges for proteins to alter pathophysiological progress. For example, circ_ZFR can promote the proliferation of hepatocellular carcinoma by binding with MAP2K1 (38). Circ_Foxo3 has high binding affinity with anti-aging ID-1, transcription factor E2F1, and anti-stress proteins FAK and HIF1A, and keeps them in the cytoplasm, leading to the aggravation of cell aging (39). ⑤ Act as a biomarker for clinical diagnosis or treatment; It is mainly due to the highly conservative nature and unique molecular structure of circRNAs, and researchers are still studying whether it can be a mature marker for the treatment and diagnosis of diseases. Therefore, the physiological characteristics of circRNAs may provide some idea for us to explore the etiology, treatment and diagnosis of DFU (**Figure 1**).

### Correlation between DFU and circRNAs

With the rapid development of the circRNA field, circRNAs in the wound tissues or blood of DFU patients has been detected by RNA-sequencing or gene microarray analysis, and the potential correlation between DFU and circRNAs expression levels has been detected. For example, Zhao et al. (40) detected circRNAs in the serum of 3 patients with DFU and 9 patients with DM, and found that compared with DM patients, the serum of DFU patients showed 10 upregulated and 23 downregulated circRNAs. Tian et al. (41) analyzed a set of microarray data and found 65 differentially expressed circRNAs, including 25 upregulated and 40 downregulated circRNAs, in 8 non-DM patients’ (normal group) tissues and 9 DFU patients’ (DFU group) tissues. Liao et al. (42) analyzed 5 non-DM patients’ tissues and 5 DFU patients wound tissues by using a set of microarray data, and found 8 differentially expressed circRNAs. All evidence indicated that circRNAs are differentially expressed in DFU and may be involved in certain academic biological processes and signaling pathways to the healing process of DFU by regulating some target genes.

## The role of circRNAs in ulcer tissue

We comprehensively summarized all circRNAs related to DFU, and found that circRNAs can regulate various cells involved in DFU wound healing, and regulate its downstream substances through some specific signaling pathways, thus acting as biological regulators (**Table 1**).

**Table 1:** Regulatory effects of different circRNAs on key genes and signaling pathways.

Authors	circRNAs	miRNAs	Targets	Signaling pathways	Function
Wang et al. (43)	circ_0084443↓	–	HBEGF/HIF1A↓	PI3K、 EGFR and ERK Signaling Pathway	Promoting proliferation and inducing migration of HEKs
He et al. (44)	circ_0084443↓	miR17-3p↑	FOXO4↓	TGFβ Signaling Pathway	Inducing migration of HEKs
Han et al. (45)	circ_PRKDC ↓	miR-31↑	FBN1/MMP2/MMP9↑	–	Inducing migration of HEKs
Jiang et al. (46)	circ_PRKDC↓	miR-20a-3p↑	RASA1↓	–	Inducing migration of HEKs
Chen et al. (47)	circ_0008450↓	–	Runx3 ↑	TGF-β/Smad signaling pathway	Inhibiting proliferation, migration and epithelial-mesenchymal transformation of HEKs
Yu et al. (48)	circ_Ttc3↑	miR-449a↓		NF-κB、PI3K-AKT signaling pathway	Raising viability and reducing apoptosis of HEKs
Zhang et al. (49)	circ_BPTF↑	miR-384↓	LIN28B↓	–	Inducing endothelial dysfunction, including apoptosis, inflammatory responses, oxidative stress
Shan et al. (50)	circ_HIPK3↑	miR-30a-3p↓	VEGFC/FZD4/WNT2↑	–	Increasing acellular capillary number
Cao et al. (51)	circ_HIPK3↓	miR-124↑	–	–	Promoting apoptosis, inhibiting migration and tube formation of ECs
Cheng et al. (52)	circ_0068087↓	miR-197↑	TLR4/NF-κB/ NLRP3↓	–	Ameliorating the inflammatory response and endothelial dysfunction of ECs
Yang et al. (53)	circ_101238↑	miR-138-5p↓	CDK6↑	–	Promoting the proliferation of FBs
Liu et al. (54)	circ_0043688↑	miR-145-5p↓	FGF2↑	–	Promoting the proliferation, migration, invasion and ECM production of FBs
Wu et al. (55)	circ_PDE7B↑	miR-661↓	FGF2↑	–	Promote the proliferation, migration and invasion of FB, and inhibit apoptosis.
Zhang et al. (56)	circ_0008259↑	–	COL1A1/COL3A1↓	–	Inhibiting collagen (II and III) synthesis
Lv et al. (57)	circ_COL5A1↑	miR-7-5p↓	Epac1↑	PI3K-AKT signaling pathway	Promote the proliferation, migration and invasion of FBs
Su et al. (58)	circ_AMD1↑	miR-27a-3p↓	COL1A1↑	–	Promoting the proliferation and collagen synthesis of FBs
Bai et al. (59)	circ_LRP6↑	miR545-3p↓	HMGA1↑	–	Promoting the proliferation, migration and invasion of VSMCs
Shi et al. (60)	circ_0008028↑	miR-182-5p↓	TRIB3↑	–	Induce proliferation, calcification and autophagy of VSMCs
Wang et al. (61)	circ_0077930 ↓	miR_622↓	KARS↑	–	Promoting senescence of VSMCs and keeps them in G1 phase for a long time
Chen et al. (62)	circ_WDR77 (circ_0013509) ↑	miR-124↓	FGF-2↑	–	Promoting the proliferation and migration of VSMCs
Liu et al. (63)	circ_ACR↓	miR-145-3p↑	–	PI3K/AKT/mTOR signaling pathway	Leading to apoptosis, autophagy, oxidative stress in SCs
Liu et al. (64)	circ_0002538 ↑	miR-138-5p↓	PLLP↑	–	Promoting the migration and myelin formation of SCs

Symbols ↑ means "Increase expression". Symbols ↓ means “Decrease expression”.

### Effect of circRNAs on human epidermal keratinocytes

Human Epidermal Keratinocytes (HEKs) are a class of epithelial cells that synthesize keratin and gradually proliferate and differentiate from deeper layers to form keratinized HEKs that act as barrier. Re-epithelization is the process of wound healing and restoration of intact epidermis, which is closely regulated by the migration and proliferation of HEKs (65, 66). Evidence suggests that migration of HEKs plays a vital role in covering the wound surface during wound re-epithelization (67, 68). Therefore, elucidating the mechanism of HEKs driving from the wound edge to the wound bed may provide crucial novel insights for the treatment of DFU. Wang et al. (43) showed that circ_0084443 was significantly expressed in DFU patients’ wound tissues and was found to be most highly expressed in HEKs, followed by FBs, ECs, and silencing circ_0084443 increased migration of HEKs, while over-expression of circ_0084443 promoted proliferation of HEKs. In addition, they found that hsa_circ_0084443 in HEKs can mediate the biological effects of PI3K, EGFR and ERK signaling pathways, but blocking these signaling pathways can inhibit the migration of HEKs (43). PI3K, EGFR and ERK signaling pathways are relatively mature and well-recognized effective pathways in the DFU healing process (69–71), which also laterally proves that circ_0084443 may play a key role in ulcer healing. SRTING database is a powerful online website for analyzing protein interactions (PPI network). Wang et al. (43) found HBEGF and HIFA in PPI network regulated by circ_0084443. Previous studies have also shown that HBEGF and HIF1A can regulate the migration and proliferation of HEKs (72–74). Furthermore, the downstream targets of circ_0084443 may not be limited to a specific one or several. For example, circ_0084443 also can inactivate TGF-β signaling pathway through miR-17-3p/FOXO4 axis, and then promote migration of HEKs (44). Han et al. (45) found that knocking down circ_PRKDC (also called circ_0084443 based on the circRNA ID of circRNA database) changes the expression of two main extracellular matrix proteins (MMP2 and MMP9) related to cell migration through miR-31/FBN1 axis, and then promotes wound healing. Jiang et al. (46) found that circ_PRKDC can directly target miR-20a-3p to regulate the expression of RASA1 and promote the migration of HEKs. This may provide strong evidence that circ_0084443 promotes the migration of HEKs. Re-epithelization requires not only the migration but also the proliferation and differentiation of HEKs. However, some studies have shown that circRNA has the biological function of regulating proliferation and differentiation. Chen et al. (47) found that circ_0008450 could activate TGF-β/Smad signaling pathway by down-regulating Runx3 expression while promoting proliferation, migration and epithelial-mesenchymal transformation of HEKs. In addition, the increase of hypoxia conditions and the damage of cells’ response to hypoxia are important reasons for the delay of wound healing (15). Yu et al. (48) found that circ_Ttc3 alleviated hypoxic injury and activated NF-κB and PI3K/AKT signaling pathways by downregulating miR-449a in HEKs. Although a small amount of circRNAs has been found in wounds today, these targets provide us with great value in the treatment of chronic wounds or in the exploration of etiological mechanisms.

### Effect of circRNAs on vascular endothelial cells

Endothelial cell (ECs) dysfunction is the initial stage of DFU. The cause of endothelial dysfunction may be due to prolonged hyperglycemia (75). Due to hyperglycemia, impaired chemotaxis, cell proliferation, and migration, the healing process is badly disturbed (14). Therefore, ameliorating endothelial dysfunction is essential for the healing of chronic wounds. It has been found that various kinds of circRNAs can regulate endothelial dysfunction. For example, Zhang et al. (49) found that high glucose (HG)-induced upregulation of circ_BPTF in ECs, and circ_BPTF regulated endothelial dysfunctions, including cell apoptosis, inflammatory responses and oxidative stress, by mediating the miR-384/LIN28B axis. Shan et al. (50) found high expression of circ_HIPK3 in HG-induced ECs, and circHIPK3 acts as an endogenous miR-30a-3p sponge to inhibit miR-30a-3p activity, thereby leading to increased expression of vascular endothelial growth factor-C (VEGFC), FZD4, and WNT2. Further study has also found that silencing circ_HIPK3 resulted in the accumulation of miR-124 and eventually endothelial dysfunction, including promotion of apoptosis, delay of the migration of ECs and inhibition of tubulation (51). A sustained HG environment promotes oxidative stress, apoptosis, and inflammatory factor expression, which result in the dysfunction of ECs (76–78). Furthermore, enhanced proinflammatory chemokines disturb wound healing, leading to diabetic ulcers (17). Cheng et al. (52) found that the down-regulation of circ_0068087 ameliorated the HG-induced TLR4/NF-κB/NLRP3 inflammasome-mediated inflammation and dysfunction of ECs by sponging miR-197. Previous studies have also shown that suppression of the NF-κB and NLRP3 inflammasome pathways ameliorate HG-induced inflammatory responses and endothelial dysfunction (79–81). This indicates that some circRNAs have regulatory effects on the well-known targets for alleviating endothelial dysfunction and inflammatory response. However, due to the lack of exploration of circRNAs, we still can’t fully know its regulatory role in ECs.

### Effect of circRNAs on fibroblasts

Fibroblasts (FBs) play an important role in wound tissue repair. They move to the wound area during wound formation and synthesize collagen and fibronectin with other extracellular matrix (ECM) to generate the forces needed to shrink the wound. Studies have shown that differential expression of extracellular matrix produced, assembled and reshaped by FBs also leads to poor wound healing in DFU (82). The latest studies have shown that circRNAs can regulate the proliferation, migration and apoptosis of FBs. Yang et al. (53) found that high expression of circ_101238 promoted the FBs proliferation *via* miR-138-5p/CDK6. FBs are the primary factors of wound healing through which they respond to the proliferation, migration, and myofibroblast differentiation capabilities of specific cytokines such as fibroblasts growth factor (FGF) (83). Liu et al. (54) found that circ_0043688 can regulate the proliferation, migration, invasion and ECM production of FBs by targeting miR- 145- 5p/FGF2 axis. Another study also showed that the circ_PDE7B/mir-661 axis accelerated the proliferation, migration and invasion of FBs by up-regulating FGF2 (55). Collagen can promote the migration of FBs to the wound area, thus accelerating wound healing and enhancing re-epithelization (84–86). Zhang et al. (56) found that over expression of circ_0008259 in FBs inhibited the production of collagen (I and III). Lv et al. (57) found that circ_COL5A1 regulates the proliferation, migration and invasion of FBs through the circ_COL5A1/miR-7-5p/Epac1 axis. In addition, circ_AMD1 can regulate p63 mutation *via* miR-27a-3p, which in turn promotes proliferation and collagen synthesis of FBs (58). Although research on circRNAs in FBs is still in its infancy, these circRNAs may still be important targets for DFU wound healing. At the same time, the above-mentioned FBs-related circRNAs were not found in DFU wounds, but their value in wound healing studies is indisputable.

### Effect of circRNAs on vascular smooth muscle cells

Vascular Smooth Muscle Cells (VSMCs), the main cells that constitute the middle membrane of blood vessels, and play an important role in various pathophysiological processes. VSMCs are known for their plasticity, which can change their morphology and growth state to exert contraction and synthesis functions (87). VSMCs exhibit a contractile phenotype under physiological conditions, and they can switch to a proliferative phenotype under extracellular stimulation (88). Studies have shown that HG may induce the excessive proliferation and migration of VSMCs, leading to vascular occlusion (89). Therefore, preventing abnormal proliferation and migration of VSMCs and promoting vascular recanalization may be a valuable direction for the treatment of DFU. It was found that circRNAs can regulate VSMCs. Bai et al. (59) found that circ_LRP6 was upregulated in HG-induced VSMCs, which promoted the proliferation, migration and invasion of VSMCs, whereas knocking down circ_LRP6 eliminated the capability of proliferation, migration and invasion *via* miR545-3p/HMGA1.Shi et al. (60) found that cic_0008028 induces proliferation, calcification and autophagy of HG-induced VSMCs *via* miR-1825P/TRIB3. In addition, Wang et al. (61) found that circ_0077930 caused senescence of VSMCs *via* miR_622-KARS and kept VSMCs in G1 phase for a long time. Chen et al. (62) found that circ_WDR77 (circ_0013509) targeted FGF-2 through miR-124 to regulate the proliferation and migration of VSMCs, while silencing circ_WDR77 played an inhibitory role.

### Effect of circRNAs on Schwann cells

Schwann cells (SCs), also known as nerve sheath cells, are myelin cells surrounding neuronal axons in the peripheral nervous system and secrete a variety of neurotrophic factors, which are closely related to peripheral neuropathy in DFU. HG-induced apoptosis and autophagy in neuroglial cells of the peripheral nervous system are considered to be the main causes for the occurrence and development of DFU neuropathy (19, 20). Liu et al. (63) observed *in vitro* that circ_ACR reduced apoptosis, autophagy and oxidative stress in HG induced SCs by downregulating miR-145-3p. And has effect on PI3K/AKT/mTOR signaling pathway. In addition, Liu et al. (64) found that downregulation of circ_0002538 expression in DFU peripheral neuropathy regulates migration and myelin formation of SCs through miR-138-5p/PLLP axis, which in turn improves symptoms. Although it provides new ideas in the pathogenesis and treatment of DFU. However, it is still necessary to conduct a comprehensive and holistic studies.

## Prospective applications of circRNAs in the diagnosis and treatment of DFU

### circRNAs as a diagnostic marker

The diagnosis of DFU is still mainly based on the clinical manifestations of patients and other auxiliary examinations, but due to the lack of typical symptoms and signs in the early stage of DFU, it is easy to be missed and misdiagnosed the disease. In recent years, with the development of gene sequencing technology, circRNA has gradually become a new exploration direction, and its potential as a diagnostic marker of DFU has been discovered. For example, the level of circ_102958 in gastric cancer tissues positively correlated with TNM staging (p= 0.032) and the area under ROC curve (AUC) was 0.74 (90). Compared with obtaining tissues, blood samples collected from DFU patients may more ethical and more acceptable. Exosomes are vesicles containing a variety of RNAs and proteins, which naturally exist in extracellular fluid and act as information exchange and substance transfer vector between cells (91). In addition, with the development of technology, it has been found that exosomes play an indispensable role in the early diagnosis, treatment and prognosis of some diseases, such as cancer and metabolic diseases (92). Chen et al. (93) analyzed a set of microarray data (GSE114248) with circ_0000907 and circ_0057362 as candidate markers and found that the AUC values of circ_0000907 and circ_0057362 in serum exosomes for the diagnosis of early stage and DM were 0.7564 and 0.8327, respectively. Meanwhile, expression of circ_0000907 and circ_0057362 was negatively correlated with ankle-brachial index (ABI) and percutaneous partial pressure of oxygen (TcPO2) (93). Based on the above research, circ_0000907 and circ_0057362 showed specificity in distinguishing DFU from diabetes in serum. Secondly, circ_0000907 and circ_0057362 in serum positively correlated with the severity of DFU, which may provide important clinical significance for Wagner stage for DFU patients in early stage. It may also be necessary to have stability and a long half-life as diagnostic markers. Han et al. (45) found that circ_PRKDC was significantly resistant to RNase R compared with PRKDC mRNA, and its half-life was longer. Zhang et al. (49) also found that RNase R hardly changed the expression of circ_BPTF, but significantly weakened the expression of linear mRNA BPTF. Although circRNA has been shown to be valuable as a diagnostic marker in DFU, it still requires strict clinical studies to detect circRNA in serum as an accurate and effective diagnostic marker of DFU. There are still some problems need to be solved, such as (a) how to select the most effective therapeutic targets; (b) evaluating the specificity and sensitivity of the screened targets; (c) specific regulatory mechanisms of targets.

### circRNA as a DFU therapeutic target

Recent studies have shown that circRNA plays an important role in DFU, and some drugs have potential binding sites with circRNA, which provides a basis for exploring drugs with circRNA as a therapeutic target. For example, Xiang et al. (94) found that circ_Krt13 and circ_Krt14 down regulated Ltga3 and Mylk4 expression through downstream miR-665-3p and miR-706 in traditional Chinese medicine (Sheng-ji Hua-yu formula) for the treatment of DFU. At the same time, this plays a key role in the inflammatory phase as well as in the maturation phase to improve the wound healing rate of DFU.

In addition, the difficulty in wound healing for DFU may be due to abnormal expression of some circRNAs, and supplementation or inhibition of some circRNAs or their downstream targets by exogenous sources may be a therapeutic method for DFU. For example, Shang et al. (95) transplanted the overexpressed circ_Klhl8 into a wound of DFU patient using ECs as a vector and found that it promoted angiogenesis by miR-212-3p/SIRT5. In addition, Cheng et al. (96) found that the expression of circ_0058092 was decreased in ECs under HG conditions, and transfection of plasmids overexpressing circ_0058092 inhibited the release of inflammatory cytokine and restored the proliferation and migration capacity of ECs *via* miR-217/FOXO3. Feng et al. (97) used PLCDH-circ_ACR-carrying lentivirus to transfect wound of DFU and found that it alleviated apoptosis, autophagy and oxidative stress in SCs by reducing miR-144-3p and thus promoting activation of PI3K/AKT/mTOR pathway.

Finally, circRNA can also be used as a key point of stem cell therapy to regulate the DFU healing process. Zhang et al. (98) found that circ_0075932 in adipocyte-derived exosome mediates AuroraA/NF-kB pathway activation by directly binding to PUM2, thereby inducing inflammation and apoptosis of HEK. However, silencing PUM2, AuroraA, or blocking NF-kB has the opposite effect. Shi et al. (99) found that exosomes derived from mmu_circ_0000250 modified adipose derived mesenchymal stem cells ADSCs promoted wound healing in DFU by inducing miR-128-3p/SIRT1-mediated autophagy. In addition, Wang et al. (100) found that circ_Gcap14 regulated miR-18a-5p/HIF-1α to enhance the expression of vascular growth factor (VEGF) in DFU (**Table 2**).

**Table 2:** Regulatory effects of different circRNAs in the treatment of DFU.

Authors	Drugs/Vectors	circRNAs	miRNAs	Targets	Signaling pathways	Function
Xiang et al. (94)	Sheng-ji Hua-yu formula	circ_Krt13↓ circ_Krt14↓	miR-665-3p↑ miR-706↑	Ltga3↓ Mylk4↑	–	Shorten inflammation period and accelerating maturation period of DFU wound
Shang et al. (95)	ECs	circ_Klhl8 (circ_0001373) ↑	miR-212-3p↓	SIRT5↑	–	Promoting angiogenesis
Cheng et al. (96)	Plasmid	circ_0058092↑	miR−217↓	FOXO3↑	–	Restore the proliferation and migration capabilities of ECs
Feng et al. (97)	Lentivirus	circ_ACR↑	miR-144-3p↓	–	PI3K/AKT/mTOR signaling pathway	Inhibiting apoptosis of SCs
Zhang et al. (98)	Exosome	circ_0075932↑	–	PUM2↑	AuroraA/NF-kB signaling pathway	Inducing inflammation and apoptosis of HEKs
Shi et al. (99)	Exosome	mmu_circ_0000250↑	miR-128-3p↓	SIRT1↑	–	Promote wound angiogenesis and inhibiting apoptosis of ECs
Wang et al. (100)	Exosome	circ_Gcap14↑	miR-18a-5p↓	HIF-1α/VEGF↓	–	Promoting angiogenesis

### Challenges of circRNAs in clinical applications

CircRNA may play different roles at different stages of DFU wound healing. For example, Han et al. (45) found that the expression level of circ_PRKDC did not decrease immediately after skin injury, but decreased by about 1.2 times on the first day after injury (inflammatory phase) and rapidly decreased about 3.4 times on the seventh day after injury (proliferative phase). This indicates that circ_PRKDC is dynamic and not static, and its role in inflammatory phase may be limited, but it plays an important role in the proliferation phase. However, many studies are still committed to studying the regulatory mechanism of circRNA as sponge of miRNA in DFU rather than studying its dynamics at various stages of wound healing. This may be due to the fact that the study of circRNAs in DFU is just beginning. The expression of circRNAs at each stage of DFU wound healing is dynamic, and there is a large number of circRNAs during DFU wound healing. It may affect specificity of circRNAs as diagnostic markers. This indicates that although the diagnostic potential of circRNA for DFU is well known, it is difficult to apply clinically due to its dynamics and instability in DFU healing process. In addition, the activities of circRNAs may overlap and the interactions are complex. CircRNAs have multiple downstream targets, regulate multiple signaling pathways, and participate in multiple physiological processes in different cells and tissues, so their functions may be different depending on the cells, tissues and target sites (43). For example, Wanni Zhao et al. (101) found that compared with the DM patients, the DFU patients had 33 circRNAs differentially expressed, of which circ_FBXO7, ATM and LMBRD1 were the most significant, and signalling pathway enrichment analysis revealed that multiple pathways such as lysosomal pathway, Chagas disease pathway, herpes simplex virus infection pathway, and methane metabolism played important roles in the development of DFU. It reveals that the expression level of circRNAs in DFU was significantly changed and a complex interaction network was formed with its downstream target gene and signaling pathway. Therefore, it is possible to act in various cells by interacting with or overlapping with different signaling pathways. For this reason, it is a great challenge to sort out the most critical circRNAs for clinical application. Although our mini review has been discussed the role of circRNAs in different cells, the current research is still not enough to clarify its role in different cells. Therefore, as the study progresses, it is necessary to conduct an objective and reasonable comprehensive analysis of the expression differences of these circRNAs in the next step.

In terms of accurate therapy, the selection of circRNAs as a therapeutic target for DFU requires an appropriate method. For example, the use of gene modification techniques or methylation enzyme modifications to alter expression level of circRNAs for therapeutic purposes, but there is a lack of reliable gene modification techniques in clinic (102). Another method is to change the expression of circRNAs in DFU tissues using circRNA mimics or viral vectors, but due to biosafety concerns, it is not suitable for clinical treatment at this stage (103). Although circRNAs are more conservative and stable than other non-coding RNAs, circRNAs are easily hydrolyzed in the wound microenvironment and enters the cell membrane in a free state with difficulty (104). This may have had little or no effect on the treatment of DFU wound healing. Therefore, if circRNAs are to be used clinically, they must be delivered in a safe, reliable, effective, and less side-effect delivery system. Unfortunately, such an effective delivery system is still lacking at this stage. Recently, several studies have shown that exosomes have the potential to become new effective delivery vectors because they can protect payloads from chemical and enzymatic degradation and escape recognition by the immune system (98, 99, 105). However, the difficulty of exosome extraction and high cost limit its clinical application and there is a lack of techniques for transferring circRNAs to exosomes at present (106, 107). Therefore, research on circRNAs is still in the basic research stage, and it is still a long way from clinical application.

## Conclusion

With the advancement of technology and deepening of study, circRNAs are expected to be accurate and effective biomarkers and targets for the diagnosis and treatment of DFU. However, the study of circRNAs is still in its infancy and is mostly based on basic research, so preclinical or clinical studies are needed to validate its future clinical application. In addition, it requires researchers to identify more specific circRNAs, and provide more evidence for diagnostic markers and therapeutic targets of DFU.

## Author contributions

DL and JG wrote the manuscript. DL, HB and XN performed the literature review and revised the manuscript. GS provided ideas and developed the protocol for the review. All authors contributed to the article and approved the submitted version.

## Funding

This study was supported by the Inner Mongolia Autonomous Region Academician Expert Workstation Construction Project (2019); Inner Mongolia People’s Hospital Doctor Research Start-up Foundation (2020BS01).

## Conflict of interest

The authors declare that the research was conducted in the absence of any commercial or financial relationships that could be construed as a potential conflict of interest.

## Publisher’s note

All claims expressed in this article are solely those of the authors and do not necessarily represent those of their affiliated organizations, or those of the publisher, the editors and the reviewers. Any product that may be evaluated in this article, or claim that may be made by its manufacturer, is not guaranteed or endorsed by the publisher.
